# CD19 CAR T‐Cell Therapy in Richter Transformation: A Multicentre Retrospective Analysis by the European Research Initiative on Chronic Lymphocytic Leukaemia

**DOI:** 10.1111/jcmm.70841

**Published:** 2025-10-22

**Authors:** Ofrat Beyar‐Katz, Ohad Benjamini, Julio Delgado, Marco Ruella, Ron Ram, Sigal Grisariu, Andrea Visentin, Elisabeth Vandenberghe, Massimo Gentile, Abraham Avigdor, Avichai Shimoni, Roni Shouval, Ronit Marcus, Stephen J. Schuster, Valentin Ortiz‐Maldonado, Guido Ghilardi, Luca Paruzzo, Tsila Zuckerman, Tamar Tadmor, Riva Fineman, Odelia Amit, Nuria Martinez‐Cibrian, Giulia Gabrielli, Batia Avni, Eva Minga, Noga Shem‐Tov, Lydia Scarfo, Ronit Yerushalmi, Arnon Nagler, Ronit Leiba, Ivetta Danylesko, Kostas Stamatopoulos, Thomas Chatzikonstantinou, Paolo Ghia, Yair Herishanu

**Affiliations:** ^1^ Department of Hematology and Bone Marrow Transplantation Rambam Health Care Campus Haifa Israel; ^2^ Ruth and Bruce Rappaport Faculty of Medicine Technion Haifa Israel; ^3^ Division of Hematology and Bone Marrow Transplantation Chaim Sheba Medical Center Ramat Gan Israel; ^4^ Gray faculty of Medical and Health Sciences Tel Aviv University Tel Aviv Israel; ^5^ Oncoimmunotherapy Unit, Department of Hematology Hospital Clinic de Barcelona Barcelona Spain; ^6^ Center for Cellular Immunotherapies University of Pennsylvania School of Medicine Philadelphia Pennsylvania USA; ^7^ Lymphoma Program, Abramson Cancer Center University of Pennsylvania School of Medicine Philadelphia Pennsylvania USA; ^8^ Bone Marrow Transplantation Unit Tel Aviv Sourasky Medical Center Tel Aviv Israel; ^9^ Bone Marrow Transplantation and Cancer Immunotherapy Department Hadassah University Medical Center Jerusalem Israel; ^10^ Faculty of Medicine The Hebrew University of Jerusalem Jerusalem Israel; ^11^ Hematology Unit, Department of Medicine University of Padua Padua Italy; ^12^ Department of Haematology St. James's Hospital Dublin Ireland; ^13^ Department of Haematology Trinity College Dublin Dublin Ireland; ^14^ Hematology Unit, Department of Onco‐Hematology Azienda Ospedaliera Annunziata Cosenza Italy; ^15^ Department of Pharmacy, Health and Nutritional Science University of Calabria Rende Italy; ^16^ Adult Bone Marrow Transplant Service, Cellular Therapy Service, Department of Medicine Memorial Sloan Kettering Cancer Center New York New York USA; ^17^ Department of Medicine Weill Cornell Medical College New York New York USA; ^18^ Unit of Hematology Bnai Zion Medical Center Haifa Israel; ^19^ Institute of Applied Biosciences Centre for Research and Technology Hellas Thessaloniki Greece; ^20^ School of Medicine Università Vita‐Salute San Raffaele Milan Italy; ^21^ Cancer Center IRCCS Ospedale San Raffaele Milan Italy; ^22^ Department of Statistics Health Care Campus Haifa Israel; ^23^ Department of Molecular Medicine and Surgery Karolinska Institute Stockholm Sweden

**Keywords:** allogeneic SCT, CAR‐T cells, Richter transformation

## Abstract

Richter transformation (RT) is a serious complication of chronic lymphocytic leukaemia (CLL), with poor outcomes. While CAR T‐cells have shown promise in large B‐cell lymphoma, their efficacy in RT remains unclear, and the role of allogeneic stem cell transplant (alloSCT) post‐CAR T‐cells has not been established. This study aimed to assess the clinical response and survival of patients with RT treated with anti‐CD19 CAR T‐cells. This retrospective multicentre study, conducted by the European Research Initiative on CLL (ERIC), included patients with RT who received anti‐CD19 CAR T‐cells between 06/2018 and 01/2024. Progression‐free survival (PFS) and overall survival (OS) were evaluated from CAR T‐cell infusion. Fifty‐four patients with RT were treated with anti‐CD19 CAR T‐cells (academic products, *n* = 29; commercial products, *n* = 25). The median age was 63 years, with 72% having an ECOG performance status (PS) of 0 to 1. Seven patients (13%) underwent alloSCT following CAR T‐cell infusion, with the indications being consolidation therapy (*n* = 4) and relapse/progression (*n* = 3). The overall response rate was 65%, with 46% achieving complete response (CR) at 1 month and 50% at 3 months. The median PFS was 8.0 months (95% CI: 2.1–13.8) and the median OS was 14.4 months (95% CI: 8.8–19.2). The median PFS was 31.6 months for patients achieving CR at 1 or 3 months post CAR T‐cells. Significant factors associated with mortality included high ECOG PS (*p* < 0.001), high LDH at CAR T infusion (*p* = 0.005), ICANS (*p* = 0.046) and no response at 1 month (*p* = 0.02). Multivariable Cox regression analysis identified treatment response at 1 month (*p* = 0.001) and increasing age (*p* = 0.5) as significant predictors of mortality. This study shows encouraging response rates and manageable toxicity for patients with RT treated with both academic and commercially available CAR T‐cell products.

## 1 Introduction

Richter transformation (RT) is a rare complication of chronic lymphocytic leukaemia (CLL), where CLL transforms into a more aggressive form of lymphoma, most commonly large B‐cell lymphoma (LBCL) [1]. RT is characterised by an aggressive clinical course and dismal prognosis. The response to conventional chemoimmunotherapy is often poor (complete response [CR] rates of about 20% to 30%) and median progression‐free survival (PFS) and overall survival (OS) are in the range of 6 and 12 months, respectively. Chimeric antigen receptor T (CAR T)‐cell therapy has shown remarkable efficacy in patients with de novo aggressive B‐cell lymphomas [2, 3, 4]. However, due to the rarity of RT and the scarcity of clinical trials, data on the effectiveness of CAR T‐cells in this setting is limited.

Recently, a multicentre retrospective study evaluated the impact of anti‐CD19 CAR T‐cell therapy in 30 patients with RT (transformation into aggressive LBCL) [5]. This study compared patients with RT to those with de novo LBCL (*n* = 283) and transformed indolent non‐Hodgkin's lymphoma (iNHL) (*n* = 141). Therapy with CAR T‐cells led to an overall response rate of 57% and a CR rate of 47% in patients with RT. Toxicity profiles were similar to those observed in other types of lymphomas. Despite these response rates, the median OS for patients with RT was significantly shorter compared to patients with de novo LBCL and transformed iNHL (9.9 months vs. 18 months vs. not reached, respectively). This indicates that while CAR T‐cell therapy can be effective in RT, its efficacy might be less favourable compared to other aggressive lymphomas. Another large retrospective analysis included 69 patients with RT treated with commercially available CAR T‐cells. In this cohort, the overall response rate and CR were 63% and 46%, respectively. Notably, patients who achieved CR had a median duration of response of 27.6 months. CAR T‐cell therapy was associated with a toxicity profile consistent with previous observations, with 16% of patients experiencing grade ≥ 3 cytokine release syndrome (CRS) and 37% experiencing grade ≥ 3 immune effector cell‐associated neurotoxicity syndrome (ICANS) [6].

The current study aims to analyse the response and survival rates among patients with RT treated with anti‐CD19 CAR T‐cells, with a specific focus on comparing different CAR T‐cell therapies (commercial and academic) and the role of alloSCT in these patients.

## 2 Methods

### 2.1 Data Collection

This is a retrospective international multicentre study, conducted by the European Research Initiative on CLL (ERIC). We gathered data from all patients with RT to LBCL who were treated with anti‐CD19 CAR T‐cells in participating centres between June 2018 and January 2024. Academic CAR T‐cells were administered in two centres; Sheba Hospital treated patients with a CD28‐based CAR T‐cell therapy targeting CD19, utilising the FMC63‐scFv, as part of a Phase I/II clinical trial (NCT02772198) [7]. The Hospital Clinic of Barcelona administered varnimcabtagene autoleucel (ARI‐0001), which employs the CD19‐A3B1 based scFv with a 4‐1BB costimulatory domain (NCT03144583) [8]. Some of these patients were previously described in other publications [5, 9, 10].

We collected data on baseline demographics; dates of CLL and RT diagnosis; immunoglobulin heavy variable (IGHV) gene somatic hypermutation status; Karyotype data by cytogenetic analysis (abnormal = 1 or more abnormalities); Cytogenetic status (chromosomes 11q, 13q, 17p and trisomy 12) by fluorescence in situ hybridisation (FISH); *TP53* gene mutation status via Sanger sequencing or next‐generation sequencing; prior CLL treatment details; treatment type; comorbidities; CAR T‐cell treatment specifics (date and type); date of last follow‐up and outcomes; CAR T‐cell‐related toxicities; and transplant‐related toxicities.

CAR T‐cell‐associated toxicity was recorded according to ASTCT consensus guidelines [11].

Institutional ethics committee approval was obtained for all participating centres.

### 2.2 Statistical Analysis

PFS and OS from the date of CAR T‐cell infusion were visualised using the Kaplan–Meier curves. Descriptive statistics were used to summarise categorical variables (frequencies and relative frequencies) and numeric variables (median and interquartile range). Univariable and multivariable analyses were conducted to identify predictors of response and survival. The factors evaluated for their correlation with treatment survival included: age, gender, comorbidities, disease status prior to CAR T therapy, lactate dehydrogenase (LDH) levels, Eastern Cooperative Oncology Group (ECOG) performance status (PS), type of CAR T product, occurrence of CRS and ICANS, infections and genetic/molecular characteristics. A receiver operating characteristic (ROC) curve analysis was performed to evaluate the predictive value of LDH and ferritin levels for mortality. The optimal cut‐off point was determined using the Youden Index, which maximises the sensitivity and specificity. Statistical significance was set at two‐sided *p* < 0.05. Analyses were performed using SPSS (V.27, SPSS Inc., Chicago, IL, USA).

## 3 Results

### 3.1 Patient Characteristics

We collected data on 54 patients with RT treated with anti‐CD19 CAR T‐cells in 10 participating centres between June 2018 and January 2024. Genetic data on CLL showed high‐risk disease characteristics: 53% (10/19) of patients had an abnormal karyotype, 56% (18/32) had del(17p), 58% (11/19) had TP53 mutations and 70% (14/20) had unmutated IGHV. The CAR T‐cell products used were: tisagenlecleucel (*n* = 20, 37%), Sheba POC (*n* = 18, 34%), ARI‐0001 (*n* = 11, 20%), axicabtagene ciloleucel (*n* = 4, 7%) and lisocabtagene maraleucel (*n* = 1, 2%) (Table 1). The median age at CAR T‐cell infusion was 63 years (range, 57–70). Prior to lymphodepletion, the ECOG PS was 0 to 1 in 39 patients (72%) and 2 to 3 in 15 patients (28%). The median number of prior CLL treatments was 2 (range, 1–3). Most patients (35/52, 67%) had received Bruton tyrosine kinase inhibitors (BTKi) previously, and 44% (23/52) had been treated with BCL2 inhibitors before RT with 36.5% (19/52) receiving both BTKi and BCL2i before RT (Table 1). According to Hans criteria, the cell of origin was classified as non‐GCB in 19 out of 23 (82.6%) patients with available data and as GCB in 4 of the 23 (17.4%) patients. FISH testing for MYC/BCL‐2/BCL‐6 rearrangements were performed in 21 patients and none were identified as double/triple hit lymphoma. All patients received at least one line of therapy for RT with a median number of prior RT treatments of 2 (range, 1–3). At the time of RT diagnosis, the majority (*n* = 41; 76%) were treated with R‐CHOP as first‐line therapy. Additionally, eight patients (14%) received either a BTKi or BCL2i), either in combination with systemic therapy or as monotherapy. Most patients (67%) received bridging therapy, with 14 patients undergoing targeted therapy (BTKi, BCL2i, monoclonal antibodies and bispecific antibodies), 12 receiving chemotherapy alone, three receiving combination therapy (chemotherapy with targeted therapy), and the remaining patients receiving steroids (*n* = 4) or radiotherapy (*n* = 3). Lymphodepletion prior to CAR T‐cell infusion included fludarabine and cyclophosphamide in 85% (46/54) of patients, and bendamustine in 15% (8/54) (Table 1). Notably, LDH levels prior to CAR T‐cell infusion were significantly higher in patients treated with academic CAR T‐cell products [274 (IQR: 222–359, min–max: 200–400) mU/mL] compared to those treated with commercial CAR T‐cell products [150 (IQR: 131–193, min–max: 120–210) mU/mL], *p* < 0.001. BTKi were not co‐administered with the CAR‐T protocol in the majority of patients (*n* = 49, 90.7%).

**TABLE 1:** Patient's characteristics.

Characteristics	All cohort, *n* = 54
Age at CAR T‐cell Infusion date, median (range)	63 (57–70.3)
Gender	
Female	17 (31.5%)
Male	37 (68.5%)
Comorbidities at diagnosis	
Yes	27 (50%)
Total lines of treatment for CLL, median (range)	2 (1–3)
Treated with BTKi at any line of treatment	35/52 (67%)
Treated with BCL2i at any line of treatment	23/52 (44%)
Total lines of treatment for Richter transformation, median (range)	2 (1–3)
Bridging therapy	36 (67%)
LDH (mU/mL) prior to CAR T‐cell, median (range)	251 (196–342)
Disease status of RT at last imaging pre CAR T‐cell infusion	*N* = 43
CR	8 (19%)
PR	10 (23%)
Stable	4 (9%)
Progressive	21 (49%)
ECOG PS at CAR T‐cell infusion	
0–1	39 (72%)
2–3	15 (28%)
Lymphodepleting regimen	
Fludarabine + Cyclophosphamide	46 (85%)
Bendamustine	8 (15%)
CAR T‐cell product type	
Tisagenlecleucel	20 (37%)
Sheba POC	18 (34%)
ARI‐0001	11 (20%)
Axicabtagene ciloleucel	4 (7%)
Lisocabtagene maraleucel	1 (2%)
Receive or continued BTKi in combination with CAR T‐cells	3 (6%)

### 3.2 Toxicity

CRS of any grade occurred in 87% (*n* = 47) of patients, with 21% (*n* = 10/47) experiencing Grade 3 to 4 CRS. ICANS was observed in 22% (*n* = 12) of patients, with high‐grade ICANS affecting 42% (*n* = 5) of these individuals. Immune effector cell‐associated HLH‐like syndrome (IEC‐HS) was reported in one patient. Infections were noted in 41% (*n* = 22) of patients, with bacterial infections being the most common (68%) (Table 2).

**TABLE 2:** Toxicity profile.

Toxicity	
CRS—*n* (%)	47 (87%)
CRS grade	*N* = 47
1	24 (51%)
2	13 (28%)
3	7 (15%)
4	3 (6%)
ICANS	12 (22%)
ICANS grade	*N* = 12
0	0
1	4 (33%)
2	3 (25%)
3	4 (33%)
4	1 (9%)
IEC‐HS	1 (1.9%)
Infection after CAR T‐cell infusion (within 30 days)	
Yes	22 (41%)
Type of infection or aetiology	
Bacterial	15 (68%)
Viral	4 (19%)
Other	3 (13%)

CAR T‐cells associated toxicities (CRS/ICANS and infections in the first 30 days) were significantly higher in academic CAR T‐cells compared to commercial CAR T products (*n* = 28, 60% vs. *n* = 19, 40%, *p* = 0.04).

### 3.3 Outcomes

**FIGURE 1:**
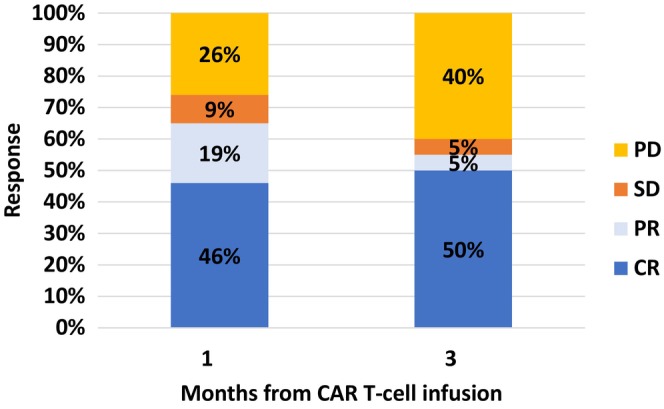
Response rates to CAR T‐cells in RT patients. Response at 1 month and 3 months post CAR T‐cells.

**FIGURE 2:**
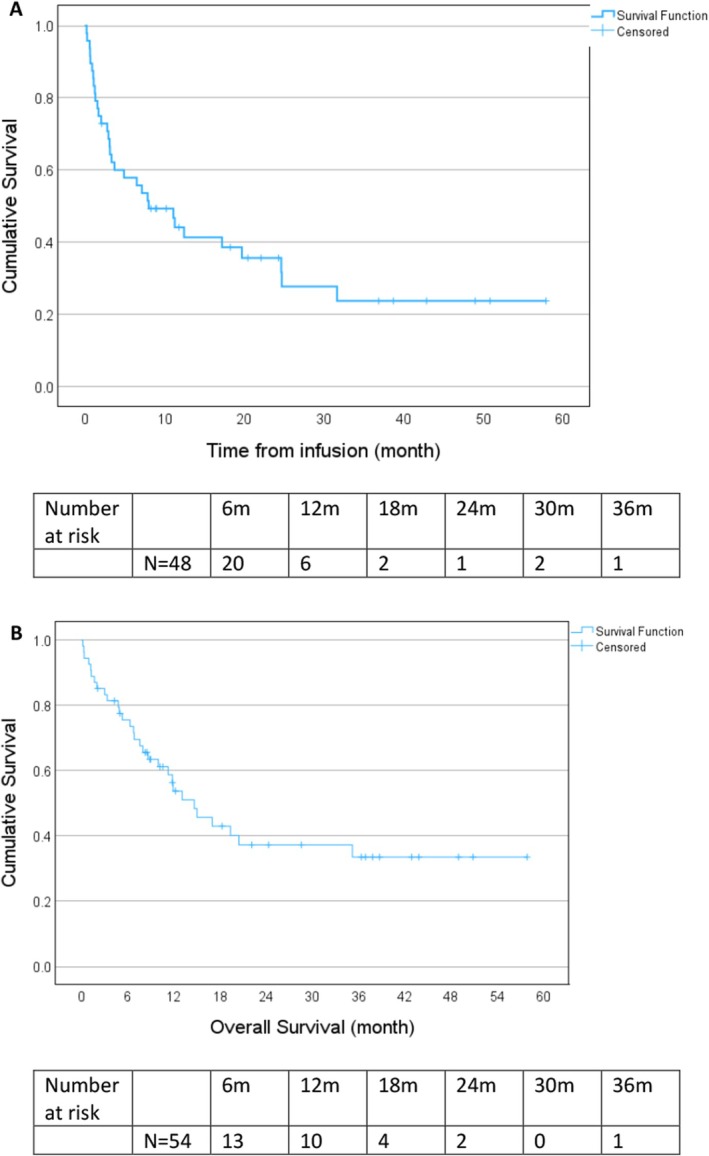
Survival of all patients. (A) Progression‐free survival in patients with RT following CAR T‐cell infusion. (B) Overall survival in patients with RT following CAR T‐cell infusion.

**FIGURE 3:**
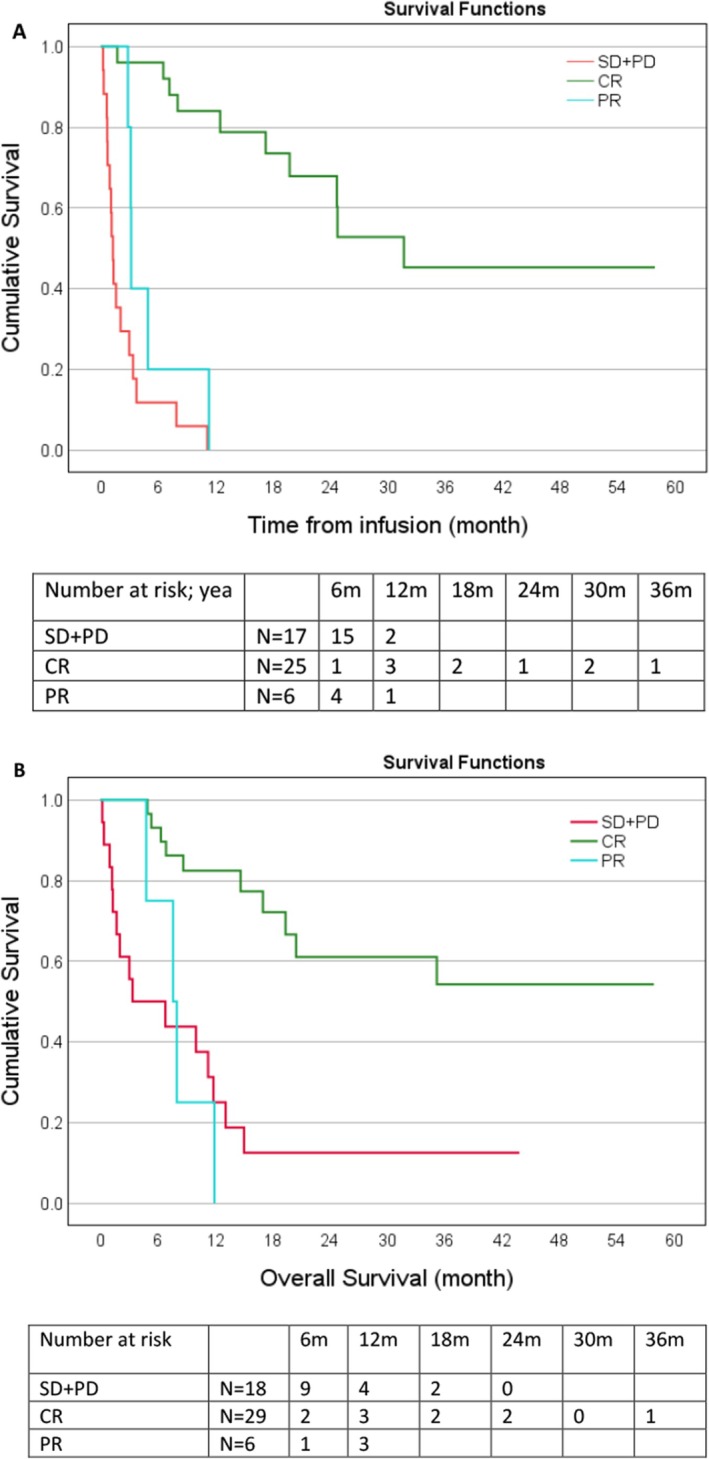
Survival based on best response to CAR T‐cell therapy. (A) Progression‐free survival of patients achieving complete response (CR), partial response (PR) and progressive disease/stable disease (PD + SD). (B) Overall survival of patients achieving CR, PR and PD + SD.

The overall response rate to CAR T‐cell therapy was 65%, with a CR achieved in 46% of patients at 1 month and 50% at 3 months post‐infusion (Figure 1). With a median follow‐up of 20.2 months (range, 2–57), PFS rates were 56% (95% CI: 42%–70%) at 6 months and 41% (95% CI: 27%–56%) at 12 months (Figure 2A). The median PFS was 8.0 months (95% CI: 2.1–13.8) and the median OS was 14.4 months (95% CI: 8.8–19.2) (Figure 2). The median PFS was 31.6 months for patients achieving CR at 1 or 3 months post CAR T‐cell compared to 1.2 months (95% CI: 0.92–1.6) for patients with stable or progressive disease (*p* < 0.001) (Figure 3A). The median OS was not reached for patients achieving CR at 1 or 3 months post CAR T‐cells compared to 3.37 months (95% CI: 0–10.7) for patients with stable or progressive disease (*p* < 0.001) (Figure 3B). There was no significant difference in response rates or mortality between the different CAR T products used.

LDH was dichotomised at a cutoff value of 239 U/L. The AUC was 0.69 (95% CI: 0.55–0.84; *p* = 0.008), indicating good discriminative ability. Univariable analysis indicated that low OS was significantly associated with a high altered ECOG PS (2–3) at the time of CAR T‐cell infusion (*p* < 0.001), high LDH at CAR T infusion (*p* = 0.005), the development of ICANS (*p* = 0.046) and no response at 1 month following CAR T‐cell infusion (*p* = 0.02) (Table 3A). Univariable Cox regression analysis identified high LDH at CAR T‐cell infusion (*p* = 0.006), development of ICANS (*p* = 0.027), lack of response at 1 month post–CAR T‐cell infusion (*p* = 0.001), and elevated ferritin levels (> 204 ng/mL) at infusion (*p* = 0.013) as significant predictors for death.

**TABLE 3:** (A) Univariable analysis for overall survival, (B) Univariable and Multivariable analysis by Cox regression model.

(A) Univariable analysis for overall survival			
	Alive, *n* = 24	Dead; *n* = 30	*p*
Age at Richter transformation	59.8 ± 10.1	62.7 ± 9.7	*p* = 0.30
	59.5 [52.5–68.8]	62 [59–69.5]	
Age at CAR T‐cell infusion	60.8 ± 10.0	64.0 ± 9.5	*p* = 0.25
	60.5 [52.8–70.8]	64 [60.8–71]	
Gender			
Female	3 (33%)	9 (30%)	*p* = 0.79
Male	16 (67%)	21 (70%)	
Comorbidities at diagnosis; yes	10 (42%)	17 (57%)	*p* = 0.27
Disease status at last imaging pre CAR T‐cell infusion	*N* = 20	*N* = 23	
CR	5 (25%)	3 (13%)	*p* = 0.38
PR	6 (30%)	4 (17%)	
Progressive	7 (35%)	14 (61%)	
Stable	2 (10%)	2 (9%)	
ECOG PS at CAR T‐cell infusion			
0	13 (54.2%)	7 (23.3%)	***p* < 0.001**
1	11 (45.8%)	8 (26.7%)	
2	0	12 (40%)	
3	0	3 (10%)	
CAR T‐cell product type			
Axicabtagene ciloleucel	3 (12.5%)	1 (3.3%)	*p* = 0.48
Lisocabtagene maraleucel	0	1 (3.3%)	
Academic	13 (54.2%)	16 (53.3%)	
Tisagenlecleucel	8 (33.3%)	12 (40.0%)	
CRS; Yes	20 (83.3%)	27 (90.0%)	*p* = 0.68
CRS grade			
I + II	16 (80%)	21 (78%)	*p* = 1.00
III + IV	4 (20%)	6 (22%)	
ICANS; yes	2 (8.3%)	10 (33.3%)	***p* = 0.046**
Infection after CAR T‐cell infusion (within 30 days)			
No	13 (54.2%)	20 (66.7%)	*p* = 0.83
Bacterial	8 (33.3%)	7 (23.3%)	
Other	1 (4.2%)	1 (3.3%)	
Viral	2 (8.3%)	2 (6.7%)	
LDH; Median + IQR	221.5 [156.3–327.3]	283 [234–402]	***p* = 0.015**
LDH CUT 239 by ROC **> = 239**	9 (37.5%)	22 (75.9%)	***p* = 0.005**
Treatment response 1 month after CAR‐T CR + PR vs. PD + SD	15/17 (88.2%)	13/26 (50%)	***p* = 0.02**
Feritin median + IQR	196 [94–311]	346 [111–854]	*p* = 0.17

B. Univariable and Multivariable analysis by cox regression model						
	Univariable			Multivariable		
	Odds ratio	*p*	95% CI	Odds ratio	*p*	95% CI
Age at CAR T‐cell Infusion date	1.02	0.23	0.98–1.06	1.06	**0.05**	1.01–1.13
Gender = Male vs. female	1.54	0.28	0.7–3.38	2.04	0.27	0.58–7.20
Treatment response 1 month after CAR‐T CR + PR vs. PD + SD	0.25	**< 0.001**	0.12–0.56	0.083	**< 0.001**	0.02–0.31
ICANS; yes vs. no	2.38	**0.027**	1.10–5.12	5.00	0.065	0.90–27.7
PET‐CT prior to CAR‐T = Disease status at last imaging pre CAR T‐cell infusion						
Progressive + Stable (REF.)						
Partial response	0.39	0.10	0.13–1.16	0.64	0.53	0.16–2.54
Complete response	0.47	0.23	0.14–1.62	2.37	0.32	0.43–12.9
LDH CUT 239 by ROC > = 239	3.29	**0.006**	1.39–7.73	0.84	0.82	0.19–3.78
Ferritin at lymphodepletion CUT > = 204	2.64	**0.013**	1.22–5.58	2.43	0.21	0.60–9.80

*Note:* All bold values have a significance number.

Multivariable analysis by Cox regression confirmed treatment response at 1 month as a significant predictor of mortality (Table 3B) in addition to increasing age (*p* = 0.05). No significant differences in response rates were observed among patients with various genetic aberrations, such as del(17p), del(13q), del(11q), trisomy 12, *TP53* mutations or IGHV mutational status (Table S1).

Notably, at 1 month following CAR T‐cell therapy, CLL response assessment showed a CR in 75% of patients, PR in 7% and disease progression in 12%.

### 3.4 Allogeneic Stem Cell Transplantation

Seven patients (13%) underwent alloSCT following CAR T‐cell infusion. The indication for transplant was consolidation therapy (*n* = 4) and relapse/progression (*n* = 3). The median PFS for patients undergoing alloSCT (from any reason) (*n* = 7) and patients not receiving alloSCT (*n* = 47) was 6.5 months (95% CI: 3–9.9) and 8 months (95% CI: 0–16), respectively (*p* = 0.46). Death was observed in 57% (4/7) of these patients, 3 due to transplant‐related toxicities and 1 due to progressive disease.

## 4 Discussion

Chemoimmunotherapy is typically the first‐line treatment for patients diagnosed with RT, although it is associated with a poor CR rate, often around 20% or lower, and a median OS of 6 to 12 months [12, 13, 14]. Alternative therapeutic approaches, such as combining targeted therapies like venetoclax with R‐EPOCH [15], have demonstrated an ORR of 62% and a 50% CR rate in first‐line RT treatment. However, for patients who do not respond to these initial therapies, novel strategies should be considered for further management.

This study adds valuable insights into the efficacy and safety of anti‐CD19 CAR T‐cells in patients with RT. The overall response rate of 65%, with 50% CR at 3 months, highlights the potential of CAR T‐cells therapy for RT. Furthermore, Grade 3 and above CRS and ICANS were observed only in 18% and 9% of patients, respectively.

Our findings align with previous research but also highlight some nuances specific to RT. Notably, Kittai et al. included patients primarily treated with axicabtagene ciloleucel, while our cohort comprised half academic‐based CAR T‐cell treatments and the remainder predominantly utilising tisagenlecleucel [6]. This represents a significant difference between the two cohorts, yet both studies demonstrate comparable response rates. Regarding toxicity, Kittai et al. reported a 37% high‐grade ICANS rate, higher than observed in the current study and in line with previous reports in the setting of LBCL with axicabtagene ciloleucel [16, 17].

Our analysis indicates that high ECOG PS, high LDH and ferritin at CAR T infusion, the development of ICANS and lack of early response are significant predictors of poor outcomes. These findings are supported by other studies in LBCL, which have shown that altered PS and severe CAR T‐cell toxicities are associated with worse efficacy outcomes in CAR T‐cell therapy [16, 18, 19, 20].

Importantly, our study confirmed also in the RT setting that the quality of the response is a strong predictor of long‐term efficacy, with patients who responded to CAR T‐cell therapy exhibiting a significantly enhanced median OS compared to those with stable disease or progressive disease (median not reached versus 3.3 months, respectively). Remarkably, achieving CR rather than PR is specifically associated with improved outcomes and durable remission. These findings align with a substantial body of literature indicating that achieving an early and deep remission is a strong predictor of prolonged survival in patients with various hematologic malignancies receiving CAR T‐cell therapy [3, 4, 17, 21, 22, 23]. Studies consistently demonstrate that patients who attain CR or a significant reduction in tumour burden shortly after CAR T‐cell infusion are more likely to experience durable responses, translating into improved survival rates.

Various genetic aberrations can predict treatment response and prognosis in CLL and RT. Increasingly, computational methods alongside comprehensive genetic, transcriptomic and epigenetic analyses are enhancing our understanding of the biology and clonal origins of RT [24]. Notably, our study found no association between treatment response and genetic aberrations (cytogenetics or FISH), *TP53* mutations or IGHV mutational status. This is consistent with prior data demonstrating robust activity of CAR T‐cell therapy in patients with high‐risk features in Diffuse large B‐cell lymphoma [DLBCL], including those with adverse genetic abnormalities [25].

The incidence of overall infections following anti‐CD19 CAR T‐cell therapy varies significantly across studies, ranging from 10% to 45% [26]. In our cohort, infections were observed in 41% of patients, placing our findings at the higher end of the reported range. This elevated rate may be attributed to the underlying immune compromise in CLL patients, as well as variations in antibacterial prophylaxis strategies across studies. These findings suggest that patients receiving CAR T‐cell therapy, particularly those with RT, may benefit from heightened vigilance and optimisation of antibiotic prophylaxis.

AlloSCT remains a debated topic in the context of RT. In our study of 54 patients, 7 (13%) underwent alloSCT following CAR T‐cell therapy. Similarly, in the study by Benjamini et al., five out of 30 patients (17%) received alloSCT, with two undergoing transplant as consolidation while in CR, and three in the setting of relapse post‐CAR T‐cell therapy [5]. In contrast, Kittai et al. [6] reported that only 4 out of 69 patients (6%) underwent consolidative alloSCT. These variations highlight the lack of consensus regarding the role of alloSCT in patients with RT treated with CAR T‐cells. Alternatively, they may reflect the general infeasibility of alloSCT in this population, often due to non‐response to CAR T‐cell therapy, advanced age, frailty or its unsuitability given the significant rates of transplant‐related mortality observed in these patients. Additionally, the small numbers do not allow any meaningful comparisons between the studies. Nevertheless, durability of response in a portion of the patients who did not undergo alloSCT may suggest that not all patients require consolidation with alloSCT.

There are several limitations to our study. First, it is a retrospective analysis with a small sample size, which may impact the generalisability of the findings. Second, as most of the participating centres were referral centres, genetic data on CLL were limited, preventing a definitive assessment of the correlation between these variables and CAR‐T cell therapy outcomes. Additionally, we lacked data on the clonal relationship between RT and CLL, as well as CLL status (including Minimal residual disease) following CAR T‐cell treatment. Lastly, the variability among commercial CAR T‐cell products introduces challenges in interpreting their risk–benefit balance at an individual level, as our findings provide limited representation of these commercial therapies.

In summary, while CAR T‐cell therapy shows clear efficacy in RT, the outcome may not be as favourable as in de novo DLBCL. Nevertheless, given the historically poor prognosis associated with RT, CAR T‐cell therapy represents a meaningful and effective treatment option for this challenging disease.

The study highlights the need for improved strategies to manage toxicities and optimise outcomes, including better patient selection, combination therapies and supportive care. Durability of response in some patients, if demonstrated in future larger trials, may challenge the role of alloSCT in RT. Future research should focus on refining these approaches and understanding the factors that influence treatment response and the role of alloSCT. Dedicated trials using CAR T‐cell in RT are also needed given the unmet clinical need in this particularly neglected setting.

## Author Contributions

**Ofrat Beyar‐Katz:** conceptualization (equal), data curation (equal), formal analysis (equal), methodology (equal), resources (equal). **Ohad Benjamini:** data curation (supporting). **Julio Delgado:** data curation (supporting). **Marco Ruella:** data curation (supporting). **Ron Ram:** data curation (supporting). **Sigal Grisariu:** data curation (equal). **Andrea Visentin:** data curation (supporting). **Elisabeth Vandenberghe:** data curation (supporting). **Massimo Gentile:** data curation (supporting). **Abraham Avigdor:** data curation (supporting). **Avichai Shimoni:** data curation (supporting). **Roni Shouval:** data curation (supporting). **Ronit Marcus:** data curation (supporting). **Stephen J. Schuster:** data curation (supporting). **Valentin Ortiz‐Maldonado:** data curation (supporting). **Guido Ghilardi:** data curation (equal). **Luca Paruzzo:** data curation (supporting). **Tsila Zuckerman:** data curation (supporting). **Tamar Tadmor:** data curation (supporting). **Riva Fineman:** data curation (supporting). **Odelia Amit:** data curation (equal). **Nuria Martinez‐Cibrian:** data curation (supporting). **Giulia Gabrielli:** data curation (supporting). **Batia Avni:** data curation (supporting). **Eva Minga:** data curation (supporting). **Noga Shem‐Tov:** data curation (supporting). **Lydia Scarfo:** data curation (supporting). **Ronit Yerushalmi:** data curation (supporting). **Arnon Nagler:** data curation (supporting). **Ronit Leiba:** data curation (supporting). **Ivetta Danylesko:** data curation (supporting). **Kostas Stamatopoulos:** data curation (supporting). **Thomas Chatzikonstantinou:** data curation (supporting). **Paolo Ghia:** data curation (supporting). **Yair Herishanu:** conceptualization (equal), data curation (equal), formal analysis (equal), methodology (equal).

## Conflicts of Interest

O.B.‐K. and R.R.—Honorarium from novartis and gilead. M.R.—Vittoria Biotherapeutics: Current equity holder in private company, Patents and Royalties; AbClon Inc.: Other: Consultancy, Research Funding. S.G.—Gilead, Medison, MSD, Novartis, Sanofi, Takeda: Consultancy. A.V.—attended scientific advisory boards organised by Johnson & Johnson, Abbvie, Astrazeneca, BeiGene and Takeda; Speaker Bordeaux for Johnson & Johnson and Abbvie. A.A.—Karyospharm: Research Funding; Ascentage: Consultancy, Honoraria, Speakers Bureau; BeiGene: Consultancy, Honoraria, Membership on an entity's Board of Directors or advisory committees, Other, Speakers Bureau; Janssen: Consultancy, Honoraria, Membership on an entity's Board of Directors or advisory committees, Other, Research Funding, Speakers Bureau; Bristol Myers Squibb: Consultancy, Honoraria, Research Funding, Speakers Bureau; A0bbVie: Consultancy, Honoraria, Membership on an entity's Board of Directors or advisory committees, Other, Research Funding, Speakers Bureau; Eli Lilly: Consultancy, Honoraria, Membership on an entity's Board of Directors or advisory committees, Other, Speakers Bureau; TG Therapeutics: Consultancy; Roche: Consultancy, Honoraria, Membership on an entity's Board of Directors or advisory committees, Other: Travel, accommodations, expenses, Research Funding, Speakers Bureau; Novartis: Consultancy, Honoraria, Membership on an entity's Board of Directors or advisory committees, Other, Research Funding, Speakers Bureau. V.O.‐M.—Pfizer: Honoraria; Kite/Gilead: Honoraria, Other: Travel grants; Miltenyi: Honoraria; Hospital Clínic de Barcelona: Current Employment; Janssen: Honoraria, Other: Travel grants; Celgene‐BMS: Honoraria, Other: Travel grants; Novartis: Honoraria. G.G.—Vittoria Biotherapeutics: Honoraria. B.A.—Sanofi: Consultancy; Johnson and Johnson: Consultancy; Novartis: Consultancy; MSD: Consultancy; Takeda: Consultancy; Medison: Consultancy. T.T.—Janssen, Roche, Abbvie, Astra, Takeda, Novartis, Beigene, Medison: Consultancy, Research Funding. L.S.—Janssen: Honoraria; Lilly: Honoraria; BeiGene: Honoraria; AstraZeneca: Honoraria; AbbVie: Honoraria; Octapharma: Honoraria. K.S.—AstraZeneca: Honoraria, Research Funding; Janssen: Honoraria, Research Funding; Novartis: Research Funding; Roche: Research Funding; Bristol Myers Squibb: Honoraria; AbbVie: Honoraria, Research Funding; Lilly: Honoraria. T.C.—Honoraria from AbbVie, AstraZeneca and BeiGene. P.G.—Loxo@Lilly: Consultancy; Galapagos: Consultancy; Johnson&Johnson: Consultancy, Research Funding; Bristol Myers Squibb: Consultancy, Research. unding; BeiGen: Consultancy; AstraZeneca: Consultancy, Research Funding; AbbvVie: Consultancy, Research Funding; MSD: Consultancy; Galapagos: Consultancy; Roche: Consultancy. O.B., J.D., E.V., M.G., A.S., R.S., R.M., S.J.S., L.P., T.Z., R.F., O.A., N.M.C., G.G., R.Y., A.N., R.L., I.D., E.M., N.S.‐T. declare no conflicts of interest.

## Supporting information


**Table S1:** Molecular and genetic data.

## Data Availability

The data that support the findings of this study are available from the corresponding author upon reasonable request.

## References

[1] T. Tadmor and I. Levy , “Richter Transformation in Chronic Lymphocytic Leukemia: Update in the Era of Novel Agents,” Cancers 13, no. 20 (2021): 5141. 34680290 10.3390/cancers13205141 PMC8533993

[2] S. S. Neelapu and A. J. C. A. Ghobadi , “2‐Year Follow‐Up and High‐Risk Subset Analysis of Zuma‐1, the Pivotal Study of Axicabtagene Ciloleucel (Axi‐Cel) in Patients With Refractory Large B Cell Lymphoma,” Biology of Blood and Marrow Transplantation 25 (2019): S65.

[3] S. J. Schuster , M. R. Bishop , C. S. Tam , et al., “Tisagenlecleucel in Adult Relapsed or Refractory Diffuse Large B‐Cell Lymphoma,” New England Journal of Medicine 380, no. 1 (2019): 45–56. 30501490 10.1056/NEJMoa1804980

[4] J. S. Abramson , M. L. Palomba , L. I. Gordon , et al., “Lisocabtagene Maraleucel for Patients With Relapsed or Refractory Large B‐Cell Lymphomas (TRANSCEND NHL 001): A Multicentre Seamless Design Study,” Lancet 396, no. 10254 (2020): 839–852. 32888407 10.1016/S0140-6736(20)31366-0

[5] O. Benjamini , S. Fried , R. Shouval , et al., “Anti‐CD19 Chimeric Antigen Receptor T‐Cell Therapy Has Less Efficacy in Richter Transformation Than in *de Novo* Large B‐Cell Lymphoma and Transformed Low‐Grade B‐Cell Lymphoma,” Haematologica 109, no. 11 (2024): 3566–3577. 38899351 10.3324/haematol.2023.284664 PMC11532690

[6] A. S. Kittai , D. Bond , Y. Huang , et al., “Anti‐CD19 Chimeric Antigen Receptor T‐Cell Therapy for Richter Transformation: An International, Multicenter, Retrospective Study,” Journal of Clinical Oncology 42, no. 17 (2024): 2071–2079. 38552193 10.1200/JCO.24.00033

[7] M. Kedmi , R. Shouval , S. Fried , et al., “Point‐of‐Care Anti‐CD19 CAR T‐Cells for Treatment of Relapsed and Refractory Aggressive B‐Cell Lymphoma,” Transplantation and Cellular Therapy 28, no. 5 (2022): 251–257. 35218999 10.1016/j.jtct.2022.02.017 PMC9519531

[8] V. Ortíz‐Maldonado , S. Rives , M. Castellà , et al., “CART19‐BE‐01: A Multicenter Trial of ARI‐0001 Cell Therapy in Patients With CD19+ Relapsed/Refractory Malignancies,” Molecular Therapy 29, no. 2 (2021): 636–644. 33010231 10.1016/j.ymthe.2020.09.027 PMC7854276

[9] V. Ortiz‐Maldonado , G. Frigola , M. Español‐Rego , et al., “Results of ARI‐0001 CART19 Cells in Patients With Chronic Lymphocytic Leukemia and Richter's Transformation,” Frontiers in Oncology 12 (2022): 828471. 35174095 10.3389/fonc.2022.828471 PMC8841853

[10] O. Benjamini , A. Shimoni , M. Besser , et al., “Safety and Efficacy of CD19‐CAR T Cells in Richter's Transformation After Targeted Therapy for Chronic Lymphocytic Leukemia,” Blood 136, no. Supplement 1 (2020): 40.

[11] D. W. Lee , B. D. Santomasso , F. L. Locke , et al., “ASTCT Consensus Grading for Cytokine Release Syndrome and Neurologic Toxicity Associated With Immune Effector Cells,” Biology of Blood and Marrow Transplantation 25, no. 4 (2019): 625–638. 30592986 10.1016/j.bbmt.2018.12.758 PMC12180426

[12] A. M. Tsimberidou , H. M. Kantarjian , J. Cortes , et al., “Fractionated Cyclophosphamide, Vincristine, Liposomal Daunorubicin, and Dexamethasone Plus Rituximab and Granulocyte‐Macrophage‐Colony Stimulating Factor (GM‐CSF) Alternating With Methotrexate and Cytarabine Plus Rituximab and GM‐CSF in Patients With Richter Syndrome or Fludarabine‐Refractory Chronic Lymphocytic Leukemia,” Cancer 97, no. 7 (2003): 1711–1720. 12655528 10.1002/cncr.11238

[13] B. S. Dabaja , S. M. O'Brien , H. M. Kantarjian , et al., “Fractionated Cyclophosphamide, Vincristine, Liposomal Daunorubicin (daunoXome), and Dexamethasone (hyperCVXD) Regimen in Richter's Syndrome,” Leukemia & Lymphoma 42, no. 3 (2001): 329–337. 11699397 10.3109/10428190109064589

[14] P. Langerbeins , R. Busch , N. Anheier , et al., “Poor Efficacy and Tolerability of R‐CHOP in Relapsed/Refractory Chronic Lymphocytic Leukemia and Richter Transformation,” American Journal of Hematology 89, no. 12 (2014): E239–E243. 25196783 10.1002/ajh.23841

[15] M. S. Davids , K. A. Rogers , S. Tyekucheva , et al., “Venetoclax Plus Dose‐Adjusted R‐EPOCH for Richter Syndrome,” Blood 139, no. 5 (2022): 686–689. 34788401 10.1182/blood.2021011386 PMC8814674

[16] C. A. Jacobson , F. L. Locke , L. Ma , et al., “Real‐World Evidence of Axicabtagene Ciloleucel for the Treatment of Large B Cell Lymphoma in the United States,” Transplantation and Cellular Therapy 28, no. 9 (2022): 581.e1–581.e8. 10.1016/j.jtct.2022.05.026 PMC9427701 35609867

[17] S. S. Neelapu , F. L. Locke , N. L. Bartlett , et al., “Axicabtagene Ciloleucel CAR T‐Cell Therapy in Refractory Large B‐Cell Lymphoma,” New England Journal of Medicine 377, no. 26 (2017): 2531–2544. 29226797 10.1056/NEJMoa1707447 PMC5882485

[18] M. Smith , A. Dai , G. Ghilardi , et al., “Gut Microbiome Correlates of Response and Toxicity Following Anti‐CD19 CAR T Cell Therapy,” Nature Medicine 28, no. 4 (2022): 713–723. 10.1038/s41591-022-01702-9 PMC9434490 35288695

[19] A. S. Kittai , Y. Huang , M. Gordon , et al., “Comorbidities Predict Inferior Survival in Patients Receiving Chimeric Antigen Receptor T Cell Therapy for Diffuse Large B Cell Lymphoma: A Multicenter Analysis,” Transplantation and Cellular Therapy 27, no. 1 (2021): 46–52. 33002640 10.1016/j.bbmt.2020.09.028

[20] L. J. Nastoupil , M. D. Jain , L. Feng , et al., “Standard‐of‐Care Axicabtagene Ciloleucel for Relapsed or Refractory Large B‐Cell Lymphoma: Results From the US Lymphoma CAR T Consortium,” Journal of Clinical Oncology 38, no. 27 (2020): 3119–3128. 32401634 10.1200/JCO.19.02104 PMC7499611

[21] M. Wang , J. Munoz , A. Goy , et al., “Three‐Year Follow‐Up of KTE‐X19 in Patients With Relapsed/Refractory Mantle Cell Lymphoma, Including High‐Risk Subgroups, in the ZUMA‐2 Study,” Journal of Clinical Oncology 41, no. 3 (2023): 555–567. 35658525 10.1200/JCO.21.02370 PMC9870225

[22] B. D. Shah , A. Ghobadi , O. O. Oluwole , et al., “KTE‐X19 for Relapsed or Refractory Adult B‐Cell Acute Lymphoblastic Leukaemia: Phase 2 Results of the Single‐Arm, Open‐Label, Multicentre ZUMA‐3 Study,” Lancet 398, no. 10299 (2021): 491–502. 34097852 10.1016/S0140-6736(21)01222-8 PMC11613962

[23] S. S. Neelapu , C. A. Jacobson , A. Ghobadi , et al., “Five‐Year Follow‐Up of ZUMA‐1 Supports the Curative Potential of Axicabtagene Ciloleucel in Refractory Large B‐Cell Lymphoma,” Blood 141, no. 19 (2023): 2307–2315. 36821768 10.1182/blood.2022018893 PMC10646788

[24] E. M. Parry , E. Ten Hacken , and C. J. Wu , “Richter Syndrome: Novel Insights Into the Biology of Transformation,” Blood 142, no. 1 (2023): 11–22. 36758208 10.1182/blood.2022016502 PMC10356575

[25] H. Shi , P. Zheng , R. Liu , et al., “Genetic Landscapes and Curative Effect of CAR T‐Cell Immunotherapy in Patients With Relapsed or Refractory DLBCL,” Blood Advances 7, no. 6 (2023): 1070–1075. 35901280 10.1182/bloodadvances.2021006845 PMC10034568

[26] E. Kampouri , J. S. Little , K. Rejeski , O. Manuel , S. P. Hammond , and J. A. Hill , “Infections After Chimeric Antigen Receptor (CAR)‐T‐Cell Therapy for Hematologic Malignancies,” Transplant Infectious Disease 25, no. Suppl 1 (2023): e14157. 37787373 10.1111/tid.14157

