# Supplementary material for: CD19 CAR T‐Cell Therapy in Richter Transformation: A Multicentre Retrospective Analysis by the European Research Initiative on Chronic Lymphocytic Leukaemia
Source: J Cell Mol Med. 2025 Oct 22;29(20):e70841. doi: 10.1111/jcmm.70841 (PMC12541669; doi:10.1111/jcmm.70841)
Supplement: Supplementary file 1 — Table S1: Molecular and genetic data. [file JCMM-29-e70841-s001.docx]

| Best response | SD+PD | CR+PR | p- value |
| --- | --- | --- | --- |
| Karyotype result  Abnormal  Normal | N=8  3 (37.5%)  5 (62.5%) | N=11  7 (63.6%)  4 (36.4%) | P=0.37 |
| del13q  negative  positive | N=9  4 (44.4%)  5 (55.6%) | N=14  9 (64.3%)  5 (35.7%) | P=0.42 |
| del11q  negative  positive | N=11  9 (81.8%)  2 (18.2%) | N=14  11 (78.6%)  3 (21.4%) | P=1.00 |
| trisomy 12  negative  positive | N=8  7 (87.5%)  1 (12.5%) | N=14  10 (71.4%)  1 (28.6%) | P=0.61 |
| del17p  negative  positive | N=12  7 (58.3%)  5 (41.7%) | N=21  8 (38.1%)  13 (61.9%) | P=0.26 |
| IGHV gene status  Mutated  Unmmutate | N=10  4 (40%)  6 (60%) | N=13  3 (23.1%)  10 (76.9%) | P=0.65 |
| TP53  Mutated  Unmmutate | N=7  2 (28.6%)  5 (71.4%) | N=13  9 (69.2%)  4 (30.8%) | P=0.16 |
